# Polo-Like Kinase 4’s Critical Role in Cancer Development and Strategies for Plk4-Targeted Therapy

**DOI:** 10.3389/fonc.2021.587554

**Published:** 2021-03-12

**Authors:** Xiaoyang Zhang, Cheng Wei, Hao Liang, Lei Han

**Affiliations:** Tianjin Neurological Institute, Key Laboratory of Post-Neuroinjury Neuro-repair and Regeneration in Central Nervous System, Ministry of Education and Tianjin City, Tianjin Medical University General Hospital, Tianjin, China

**Keywords:** polo-like kinases family, cancer development, inhibitors, centrosome amplification, PLK4

## Abstract

Polo-like kinases (Plks) are critical regulatory molecules during the cell cycle process. This family has five members: Plk1, 2, 3, 4, and 5. Plk4 has been identified as a master regulator of centriole replication, and its aberrant expression is closely associated with cancer development. In this review, we depict the DNA, mRNA, and protein structure of Plk4, and the regulation of Plk4 at a molecular level. Then we list the downstream targets of Plk4 and the hallmarks of cancer associated with these targets. The role of Plk4 in different cancers is also summarized. Finally, we review the inhibitors that target Plk4 in the hope of discovering effective anticancer drugs. From authors’ perspective, Plk4 might represent a valuable tumor biomarker and critical target for cancer diagnosis and therapy.

## Introduction

Centrosomes are recognized as microtubule-organizing centers (MTOCs) during mitosis in most eukaryotic cells. Two tubular structures called centrioles are connected proximally to form centrosomes (1–3). Centrosomes are involved in many activities that are important to cellular physiological functions, such as cell movement and cell division (4–6). Hence, it is necessary to strictly control the replication of centrosomes (7, 8). Centrosome amplification has a close association with chromosomal instability (CIN), which causes tumorigenesis and poor clinical outcomes (9–11). Thus, investigating the role of the centrosome in tumorigenesis and cancer progression is of great significance.

**Figure 1:**
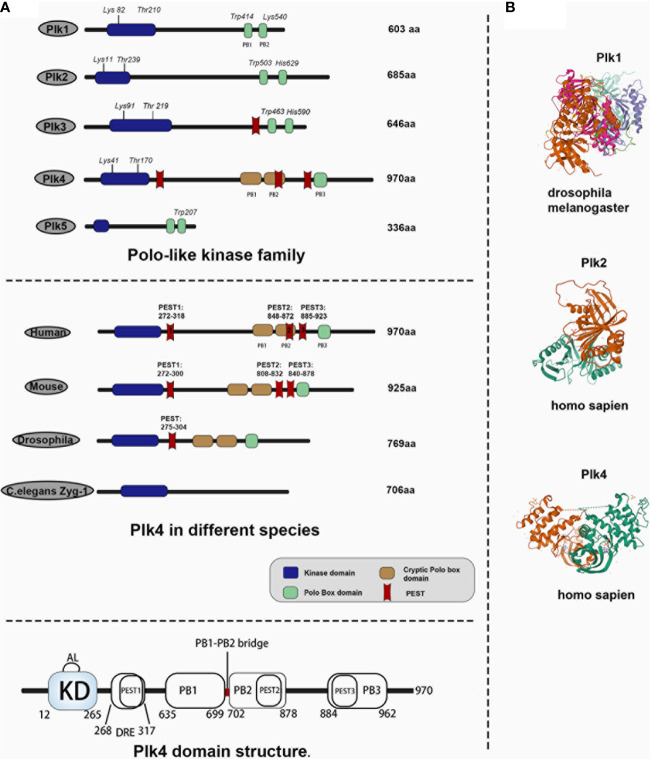
Comparison of Plk4 protein structure and crystal structure with other Plk family members in human and Plk4 structure in other species. **(A)** Upper panel, protein structure of Plk family members in human species. Middle panel, protein structure of Plk4 in different species. Lower panel, schematic representation of Plk4 domain structure. **(B)** protein crystal structure of Plk1 in *drosophila*, Plk2 and Plk4 in *homo sapiens*.

Mammalian polo-like kinases (Plks) are essential regulators of the cell cycle, centriole duplication, mitosis, cytokinesis (12, 13). All Plks, except for Plk5, contain a catalytically active kinase domain (KD) located at the N-terminal, and a polo-box domain (PBD) at the C-terminal. The KD determines the kinase activity, and PBD is critical to binding substrates and regulates their kinase activity (14–17). There is an ATP-binding site in KD that can be targeted by ATP-competitive Plks inhibitors to exert inhibiting effects (18, 19). The PBDs, which have not been found in other proteins (20), determine the substrate specificity of Plks kinases through protein‒protein interaction and regulate their function (21, 22). The functions of the five Plk family members (Plk1–5) were all identified in mammalian cells (16). Plk1 is important for centrosome separation and maturation (23, 24), mitotic entry (25), spindle formation (26), chromosome segregation (27, 28), and cytokinesis (29); Plk2 and 4 are implicated in centriole duplication (10, 30); Plk3 is critical in DNA replication (31) and mediates cellular stress (13); and Plk2 and Plk5 are involved in neuron differentiation (32–34). Structural differences between Plk1‒5 are shown in **Figure 1A**, upper panel. Crystal structures of Plks for different species are illustrated in **Figure 1B**.

Plk1, 2, 3, and 5 contain two PBs (PB1 and PB2), both of which are located at the C-terminal, whereas Plk4 contains three polo-boxes (PB1, PB2, and PB3), and only PB3 is located at the C-terminal (12, 35, 36). Derived from Plk1 (37), Plk4 is a master regulator of centrosome amplification and impacts mitotic progression (38–41). Plk4 dysregulation is a main cause of mitotic catastrophe, including chromosomal mis-segregation and cytokinesis failure, which is closely related to tumorigenesis and progression (42–46). Thus, Plk4 has received the priority concern because it is recognized as a bridge between centrosomes and cancer.

## Polo-Like Kinase Family Members

**Figure 2:**
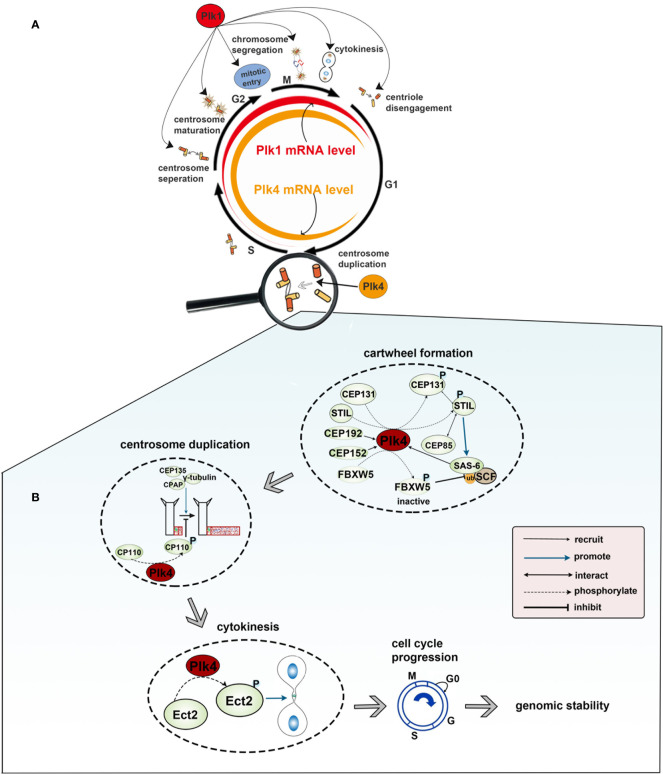
**(A)** Functions and expression level of Plk1 and Plk4 throughout the cell cycle. Colored rings in the middle represent dynamic change of Plk1 and Plk4 mRNA expression level in the cell cycle in which the width of the red and yellow band represents the expression level of Plk1 and Plk4, respectively. The outer circle represents the function of Plk1 and Plk4 in the cell cycle. **(B)** Role of Plk4 in maintaining genomic stability *via* regulating centriole biogenesis.

There are five members of polo-like kinase family: Plk1(16p12.3), Plk2 (5q12.1–13.2), Plk3 (1p34.1), Plk4 (4q27–28) and Plk5 (p13.3). And the localization of Plk1 is changing throughout the cell cycle. Plk1 usually gathers in the centrosome of the spindle poles in early period of mitosis, and then migrates gradually from spindle poles to the equatorial plate after entering into middle and late period of mitosis. At the end of mitosis, Plk1 gathers in the midbody. In mammals, Plk1 expression is elevated in actively proliferating cells and is significantly different among the different stages of the cell cycle. Plk1 expression is barely detectable in G1 and S phase, gradually increases in G2 phase, and peaks in M phase. After the completion of cell division, Plk1 expression would get a sharp decline and then move into the next loop of cell cycles (**Figure 2A**). Plk1 is a key player in cell cycle progression (12, 23, 24). Activated Plk1(p-Thr-210) at the centrosome as well as Plk1-mediated phosphorylation of pericentrin in human cells are essential for centrosome maturation in G2 (47, 48). Plk1 is the part of the positive feedback loop that activates M phase-promoting factor (MPF) by activating CDC25 (49) and by inhibiting both MYT1 and WEE1 (50), which promotes entry into mitosis in a normal cell cycle (25). In prophase, Plk1 is recruited to the chromosome arms, where it promotes the release of the cohesin complex in order to ensure accurate chromosome segregation (51). In anaphase, Plk1 undergoes extensive re-localization from the centrosome and kinetochores to the spindle midzone, where it functions as a platform for the coordinated recruitment of numerous signaling proteins that regulate cytokinesis (29). Lastly, Plk1 and separase trigger the centriole disengagement at the exit from mitosis and is required for centrioles to duplicate in the next cell cycle (12) (**Figure 2A**). Plk2, which localizes at or near the centrioles throughout the cell cycle, plays a role in regulating centriole duplication by binding to centromere protein J (CENPJ), which is involved in controlling centriole size and PCM recruitment (30, 52). Besides, FBXW7, the ubiquitin ligase targeting cyclin E for degradation, is degraded by Plk2, which results in cyclin E accumulation and promotes centriole duplication at the G1/S transition (53). Plk2, as well as Plk5, could influence neuronal activity. Plk2 activation during neuronal activity affects the regulation of synapses in proximal and distal dendrites (36). And for Plk5, it consequently lacks kinase activity and is mainly expressed in the brain, where it has been demonstrated to have a role in the formation of neuritic extensions of neurons (16). Located to the nucleolus, Plk3 interacts with and promotes the accumulation of cyclin D and cyclin E. These interactions lead to the activation of the key cell cycle protein phosphatase CDC25A, promoting DNA replication (31). Additionally, Plk3 is a stress response protein and is activated under various stress conditions, such as DNA damage, oxidative, mitotic, hypoxic and hyperosmotic stress. The expression level of human Plk4 is undetectable at the G0 phase, increases at the G1/S phase, remains at the later M phase, and finally decreases at the early G1 phase. It localizes to the perinuclear region in G1, to the nucleolus and peri-nuclear region in G2/M. In prophase, Plk4 is positioned in the centrosomes; when the cell cycle progresses to the anaphase, Plk4 is localized to the whole cell, and then to the cleavage furrow at the telophase. It has been confirmed that Plk4 is a master regulator in centriole-independent MTOC formation (**Figure 2A**).

## The Regulation of Plk4 at a Nucleic Acid Level

### Plk4 DNA and mRNA

The human Plk4 gene was mapped onto chromosome 4q27–28 and has four transcript variants (variants1‒4). Variant 1 is the longest, comprised of 17 exons, and encodes a 970-aa Plk4. Exons 4 and 1 were found to be missing in variants 2 and 3, respectively. Variant 4 only encodes a 105aa protein, which is much shorter than the protein products encoded by the other three variants (54). The transcriptional level of human Plk4 is undetectable at the G0 phase, increases at the G1/S phase, remains at the later M phase, and finally decreases at the early G1 phase (55, 56) (**Figure 2A**). At a transcription level, Plk4 can be regulated by its upstream molecules.

### Transcriptional Regulation of Plk4

**Figure 3:**
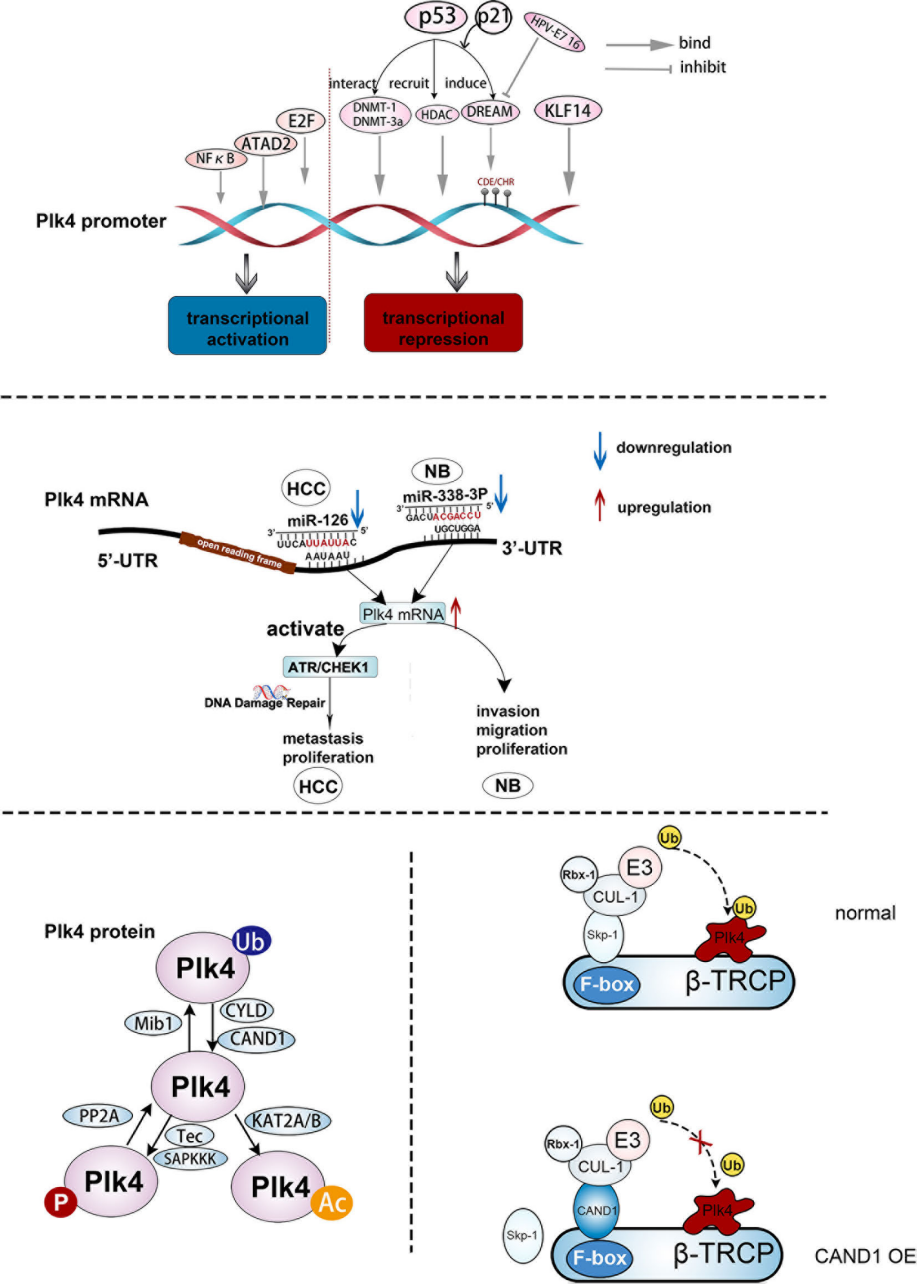
The regulation of Plk4 at DNA, mRNA, and protein levels. Upper panel, upstream effector molecules that can act on the Plk4 promoter and exert transcriptional activation and inhibition effect respectively. Middle panel, two microRNAs (miR-126 and miR-338-3p) bind to different regions of 3 ‘-UTR of Plk4 mRNA to play a role in post-transcriptional regulation of Plk4. Lower left protein translational modification of Plk4 about phosphorylation, ubiquitination and acetylation. Lower right, mechanism by which CAND1 competitively inhibits Plk4 ubiquitination degradation.

Plk4 transcription could be activated or repressed by a number of transcription factors by modulating Plk4 promoter activity (**Figure 3**, upper panel). Dysregulation of cellular transcription factor E2F has been linked to cancer progression (57, 58). A previous study on breast cancer showed that elevated Plk4 transcript levels were strongly correlated with E2F overexpression. Further analysis suggested that E2F could directly bind to the Plk4 promoter between exons 1 and 2 and increased its promoter activity, resulting in Plk4 overexpression and centrosome amplification, which eventually leads to genomic instability (GIN) and tumorigenesis (59). In addition, nuclear factor kappa B (NFκB) regulates cell cycle-related genes so as to affect many cellular processes (60). The Plk4 was found to be one of its targets. In U2-OS cells, Plk4 mRNA was downregulated upon knockdown of NFκB. Moreover, direct binding of Plk4 promoter and NFκB subunits was proved in both U2-OS and Hela cells. Thus, E2F and NFκB are transcriptional activators of the Plk4 (61). The ATPase family AAA domain-containing protein (ATAD2) (62) was also found to interact with the Plk4 promoter during Plk4 transcription (63). Wang et al. demonstrated that in U87 cells, ATAD2 activated the transcription of Plk4, and overexpression of ATAD2 caused Plk4 upregulation in U87 cells, which has the same consequence as E2F (63) (**Figure 3**, upper panel).

The Plk4 could also be transcriptionally repressed. Loss of Krüppel-like factor 14 (KLF14) gene had been identified as a link between centrosome amplification and tumorigenesis in mice (64). Fan et al. showed the absence of the KLF14 upregulated Plk4 at the mRNA and protein levels in mouse embryonic fibroblasts (MEFs). Additionally, several possible motifs within the Plk4 promoter that bind to KLF14 were discovered from the TRANSFAC database, and the binding of KLF14 to the Plk4 promoter was further confirmed in Hela cells. Thus, KLF14 transcriptionally repressed Plk4 and played a role in preventing centriole duplication in human cancer cells (64). In addition, p53 was involved in the transcriptional repression of Plk4 *via* various pathways. When DNA was damaged, the DREAM complex, which contains DP, RB-like, E2F4, and MuvB, was formed due to the activation of p53 and p21, causing transcriptional repression of Plk4 *via* binding to CDE/CHR sites of the Plk4 promoter (65). In addition, p53 interacted with the DNA methyl transferases (DNMT) DNMT1 and DNMT-3a, resulting in the hypermethylation of the Plk4 promoter and a decrease in the Plk4 expression level (66). Aside from direct binding to the promoter, the acetylation of the Plk4 promoter reduces its transcription level (67). Li et al. suggested that p53-mediated recruitment of histone deacetylase (HDAC), a transcriptional repressor, could repress Plk4 mRNA expression by removing the acetyl group from the Plk4 promoter (68) (**Figure 3**, upper panel).

### Post-Transcriptional Regulation of Plk

After Plk4 is transcribed into mRNA, the regulation still takes places, in which non-coding RNAs (ncRNAs) would play crucial roles (**Figure 3**, middle panel). MicroRNAs can specifically combine with their target mRNAs *via* binding to the 3′ untranslated region (3’-UTR) (69–71), leading to mRNA destabilization (72). MiR-126 and miR-338-3p were found to specifically bind to different sites in the 3’-UTR region of Plk4 mRNA, leading to its destabilization. These processes were observed in the Hep3B and SMMC7721 HCC cell lines and the SK-N-SH and SK-N-AS neuroblastoma cell lines (73, 74).

## Regulation of Plk4 at Protein Level

### Plk4 Protein

Human Plk4 protein (109~kDa) contains 970 amino acids (75, 76). Unlike other Plk family members, Plk4 has an extra PB3, and its PB1 and PB2 can form a homodimer. As described above, there are three PBDs in Plk4, namely PB1, PB2, and PB3. Unlike the PB1 and PB2 of other Plk family members, the PB1 and PB2 of Plk4 are special in terms of both their behavioral and biological functions. It has been reported that PB1 and PB2 of Plk1‒3 would form an intramolecular heterodimer which is required for the activity to phosphorylate and interact with their substrates (22). However, PB1 and PB2 of Plk4 form a cryptic polo-box (CPB), which in turn forms a homodimer with another of the CPBs of Plk4, thus creating a platform for Plk4 to interact with other molecules. For example, Plk4 would interact with and localize to CEP192 and CEP152 at subcentrosomal structures (77–79). Notably, the PB3 of Plk4 was not found in other Plks. PB3 of Plk4 is essential for regulating Plk4 kinase activity by relieving the autoinhibition caused by linker 1 (L1) of Plk4, although PB3 is not necessary for forming a Plk4 dimer, but Plk4 lacking PB3 exhibits greater dimerization (80). And previous study also demonstrated that the coiled-coil region (CC region) of the SCL/TAL1 interrupting locus (STIL) protein directly binds to PB3 of Plk4, which is important for regulating centriole duplication (81). In addition to PBs, Plk4, unlike other Plk family members, has three PEST motifs (rich in proline (P), aspartate (D), glutamate (E), serine (S), and threonine (T) residues) (82), which were thought to be associated with reduced protein stability (55, 83, 84). Knockdown experiments had demonstrated that all these three motifs were involved in regulating of Plk4 protein stability, while the first PEST sequence (aa272‒318) was found to be more effective than the other two sequences located at aa808‒832 and aa840‒878 (76, 85, 86) (**Figure 1A**, middle panel). Two linker regions, L1 and L2 and a PB1‒PB2 bridge were also discovered in the Plk4 protein (**Figure 1A**, lower panel), and modifications of these regions are important in auto-regulation (see below). **Figure 1A**, middle panel shows that the Plk4 proteins in mouse and Drosophila share a very similar domain structure to that in humans, whereas ZYG-1, the Caenorhabditis elegans homologue of Plk4, lacks sequence similarity to the Plk4 protein structures of other species. The Plk4 protein was also regulated at the post-translation level (see below).

### Auto-Regulation of Plk4

**Figure 4:**
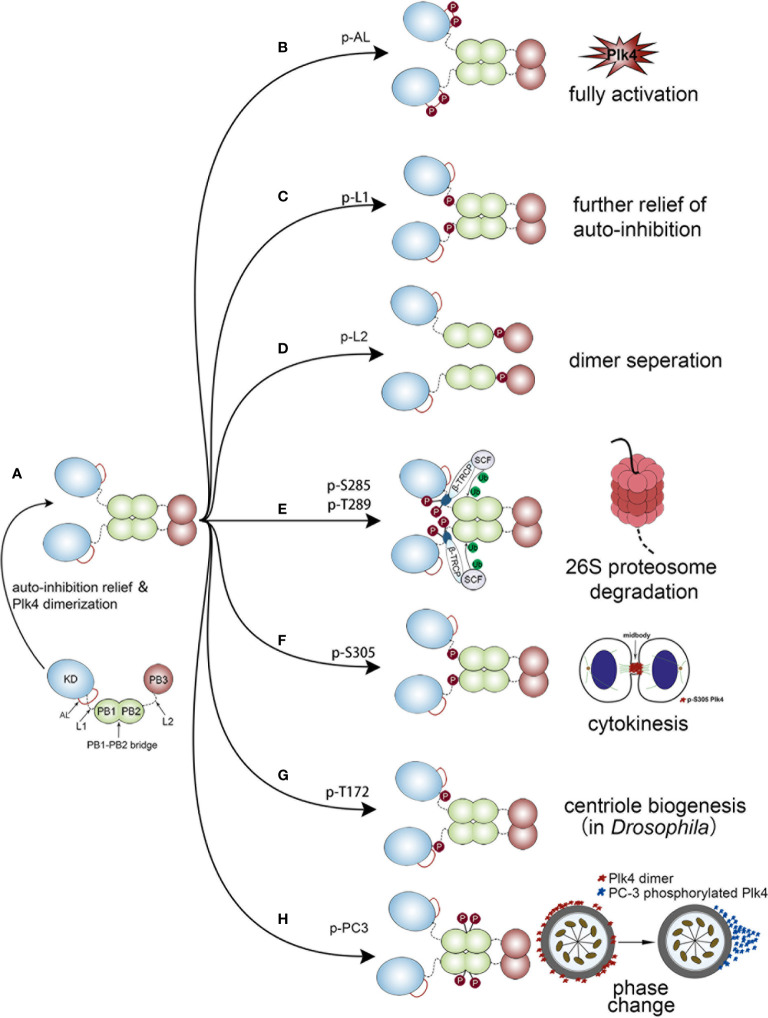
Different biological functions of Plk4 protein when phosphorylated at different sites. The left panel of the figure shows the monomer Plk4 form a polymer dimer by separating AL and L1. And the right panel shows seven different types of autophosphorylation and the corresponding biological functions. **(A)** The generation of a Plk4 homodimer. **(B)** Phosphorylation of AL leads to fully activate Plk4 activity. **(C)** L1 phosphorylation further relieves autoinhibition. **(D)** Phosphorylation of L2 causes dimer seperation. **(E)** Phosphorylation of DRE leads to ubiquitination of the PB1 and subsequent 26S proteasome mediated Plk4 degradation. **(F)** Phosphorylation of Ser-305 of Plk4 is critical for cytokinesis. **(G)** Trans-autophosphorylation of Drosophila Plk4 Thr172 critical for centriole formation. **(H)** PC3 auto-phosphorylation is important for phase change of Plk4 in centriole duplication.

Upon completion of protein synthesis, Plk4 is in a L1-mediated autoinhibited monomolecular state, in which L1 prevents the phosphorylation of the activation loop (AL) of the KD. As a result, the monomeric Plk4 protein is in an auto-inhibitory status and its kinase activity is also significantly reduced. Subsequently, PB1 and PB2 mediate the generation of a Plk4 homodimer, and then PB3 relieve autoinhibition *via* separating L1 from AL (**Figure 4A**). Thus, this homodimerization state is necessary for releasing auto-inhibition and stimulating the kinase activity (80). Based on the dimerization state, several domains of Plk4 would be phosphorylated, leading to different cellular processes.

Phosphorylation of AL leads to fully activate Plk4 activity (**Figure 4B**). L1 phosphorylation further relieves autoinhibition (**Figure 4C**), by causing the disintegration of the high-order Plk4 complex (80) (**Figure 4D**).

Plk4 protein degradation was mediated by auto-phosphorylation of downstream regulatory element (DRE) (human: aa282‒305), located within the L1 region (**Figure 1A** lower), in which 13 serine/threonine residues were identified as critical phosphorylation sites (87, 88). Among them, phosphorylation of Ser285 is necessary for Plk4 protein degradation, while Thr289 phosphorylation facilitates this process (89). The same phenomenon was also observed in Drosophila in which the corresponding residues are Ser293 and Thr297 (90). Upon auto-phosphorylation of DRE, SCFβ-TRCP E3 ubiquitin ligase complex, consisting of SKP1, CUL1, RBX1, and F-box protein (86, 91), would bind to these phosphorylated residues, leading to ubiquitination of the PB1 and subsequent 26S proteasome mediated Plk4 degradation (85, 86) (**Figure 4E**). Aside from Ser285 and Thr289 in DRE, Ser305 was also found to be phosphorylated. Sillibourne et al. showed that auto-phosphorylation of Ser305 lead to kinase activation (88) and thus, the active fraction can be recognized. Unlike Ser285 and Thr289, autophosphorylation of Ser305 (p-Ser305) does not control the stability of Plk4 (43, 88). Press et al. showed that location of p-Ser305-Plk4 is constantly changing. At metaphase and anaphase, it was detected at the kinetochores, then transferred to the midzone at anaphase and telophase, and finally, during cytokinesis, it localized to the midbody. Thus, it is assumed that p-Ser305-Plk4 is required for cell division (92) (**Figure 4F**).

In addition, Plk4 kinase activity was also regulated by auto-phosphorylation. Lopes et al. discovered that trans-autophosphorylation of Drosophila Plk4 Thr172 within the L1 motif was critical for Plk4 kinase to exert a biological function in centriole formation (93) (**Figure 4G**). Moreover, a PC3 motif (aa:698‒707), located at the PB1‒PB2 bridge (aa:700–701), was also found to be auto- phosphorylated and plays a critical role in centriole biogenesis. Park et al. discovered that auto-phosphorylation of PC3 transformed it from a ring to a dot state of Plk4. Then the dot state of Plk4 was targeted for STIL-HsSAS6 loading (94). Previous studies demonstrated that the dot state of Plk4, STIL and SAS-6 would unite into a restricted region on the CEP152/CEP195 ring which is located at the distal end of the mother centriole. This region is where the procentriole assembles (95, 96). Collectively, these findings indicate that the spatial pattern formation of Plk4 caused by PC3 auto-phosphorylation is important for centriole duplication (94, 97) (**Figure 4H**).

### Phosphorylation and Dephosphorylation of Plk4

In addition to autophosphorylation, Plk4 was phosphorylated and dephosphorylated by upstream regulators. Reflecting a variety of cellular stresses, mammalian somatic cells generate several SAPKKKs (such as MTK1, TAK1, and MEKK1) that regulate cell fate. It has been shown that these SAPKKKs regulate Plk4 activity by phosphorylation of Plk4 on Thr170. Additionally, Yamashita et al. discovered that Tec, a protein tyrosine kinase, increased Plk4 concentration and stability *via* phosphorylation of the tyrosine residue of Plk4 in HEK293 cells. While the underlying mechanism remains elusive, a possibility is that the phosphorylation of the Plk4 tyrosine residue by Tec results in a change in the Plk4 protein structure and impairment of the ubiquitination mediated Plk4 degradation (76, 98) (**Figure 3**, lower, left).

As discussed above, Plk4 auto-phosphorylation on DRE site is critical to its degradation (98). Previous studies found that protein phosphatase 2A (PP2A) is involved in regulation of the Plk4 protein level. The PP2A regulatory subunit TWS dephosphorylates and neutralizes Plk4 autophosphorylation, and thus stabilizes Plk4 and promotes centriole duplication (99) (**Figure 3**, lower, left).

### Ubiquitylation and Deubiquitylation of Plk4

Accumulating evidences indicates that ubiquitin modification alters substrate interaction and have an impact on different cellular activities including proteasomal degradation, protein activity, and protein‒protein interactions. Deubiquitinating enzymes (DUB) can reverse ubiquitination by removing ubiquitin from the substrate (100).

The cylindromatosis tumor-suppressor gene (CYLD) has been identified as a DUB and could interact with Plk4. It has been shown that the USP domain of CYLD binds the PUB domain of spermatogenesis-associated protein 2 (Spata2) (101), leading to recruitment of CYLD to the centrosome and de-ubiquitination of Plk4. Subsequently, the deubiquitinated Plk4 binds to and phosphorylates NEK7 at Ser204, which results in a weakening of the interaction between NEK7 and NLRP3. This process is necessary for NLRP3 inflammasome activation (102). Additionally, ubiquitin ligase was also recognized as an important partner in the modification of Plk4. Cajanek et al. showed that the E3 ligase activity of Mib1 triggers ubiquitylation of Plk4 on multiple sites, resulting in the formation of Lys11-, Lys29-, and Lys48- ubiquitin linkages. Lys11 and Lys48 linkages are associated with fast proteasomal degradation of Plk4 whereas Lys29 linkage impairs the binding capacity of Plk4 with CEP152 and CEP192, leading to a reduction of its association with centrioles (103). Therefore, Mib1 regulates the density of Plk4 and its interaction with centrosomal proteins, thus neutralizing the centriole amplification mediated by upregulated Plk4 level. In addition, CAND1 can adjust SCF E3 ubiquitin ligase-mediated Plk4 ubiquitination *via* its binding to CUL1 (104). Korzeniewski et al. found that CAND1 stabilized Plk4 by direct interaction with CUL1 to exclude SKP1 from the SCF complex. Thus, Plk4-mediated centriole amplification was enhanced by upregulation of CAND1 in human prostate cancer cells (20, 104) (**Figure 3**, lower, right).

### Acetylation of Plk4

Lysine acetylation is a reversible process that can regulate the cellular pathways inside and outside the eukaryotic nucleus. Lysine acetyltransferases (KATs) is an important group of enzymes that regulate this process (105). KAT activation had been observed in many diseases, especially cancer (106). Fournier et al. demonstrated that KAT2A/2B acetylates Plk4 on residues Lys45 and Lys46, and the acetylated form of Plk4 (Plk4ac) co-localized with KAT2A/B at the centrosome in the G1/S phase and possessed lower kinase activity. When KAT2A/2B was downregulated, Plk4ac decreased and Plk4 kinase was activated, leading to centriole over-duplication (107) (**Figure 3**, lower, left).

## Plk4 With Hallmarks of Cancer

**Figure 5:**
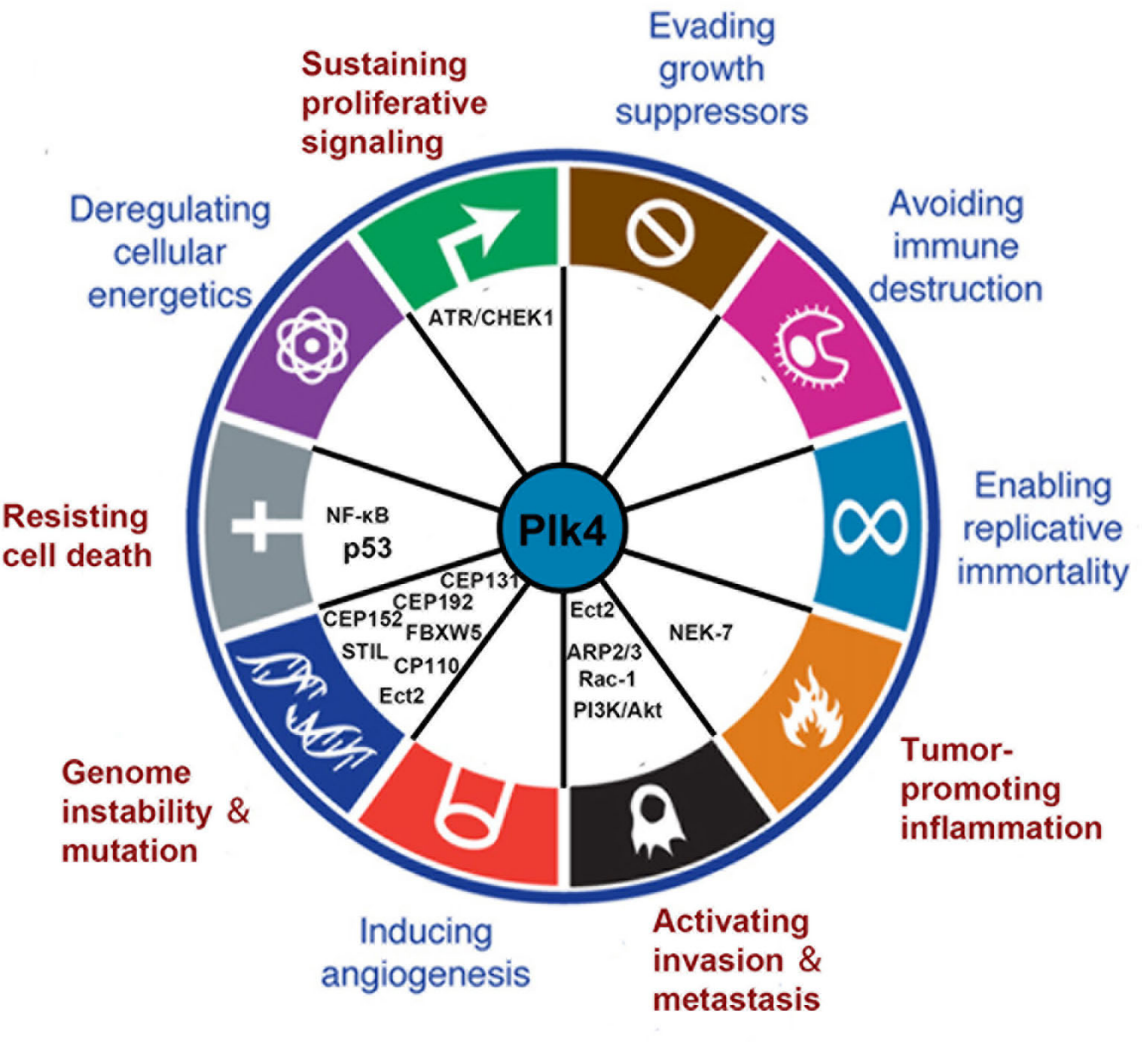
Connections between Plk4 and the hallmarks of cancer. Five hallmarks were identified (written in red). The corresponding Plk4 downstream molecules (written in black) are filled in the blank.

Plk4 dysregulation was found to be related to the onset and progression of various cancers. Thus, Plk4 has emerged as a pivotal player in cancer development. In this section, we summarize recent studies on the correlation between Plk4 and the hallmarks of cancer (108) (**Figure 5**).

### Plk4 in Genome Instability

Genomic instability (GIN) is closely associated with cancer development (109). The chromosomal instability (CIN), caused by mitotic errors, is a form of GIN that involves frequent cytogenetic changes and aberrant chromosome copy numbers (110, 111). Mitotic errors includes abnormalities in centrosome copy number, cell cycle regulation and mitotic checkpoint function (112). There is only one-time centrosome amplification per cell cycle, and Plk4 is critical to the whole process (113, 114). Plk4 was initially recruited to the ring state CEP192 and/or CEP152, which is located at the end of the parent centriole (77, 79, 115, 116). And CDK11p58 and CEP78 are critical in regulating Plk4 recruitment; the depletion of either these two proteins cause Plk4 recruitment failure (117, 118). After recruiting Plk4, the coiled-coil domain of STIL binds to the PB3 domain of Plk4, leading to Plk4 phosphorylation of STIL on the STAN motif and subsequent SAS-6 recruitment (81, 95, 119). The interaction of these two proteins forms a nine-fold symmetric cartwheel structure for centrosome replication (120, 121). In addition, Plk4 was shown to phosphorylate CEP131 on S21 and T205, and p-CEP131 interacted with STIL and recruits it to the centriole. Moreover, the Plk4 protein was also stabilized because CEP131 overexpression mediated excessive recruitment of STIL. Thus, these events lead to a higher level of Plk4 and subsequent centriole overduplication (122). CEP85 was also found to be required for STIL localization. CEP85 interacts with STIL resulting in Plk4 activation and centriole assembly (123). Moreover, FBXW5, a component of the SCF complex, was phosphorylated and inactivated by Plk4. Inactivated FBXW5 reduces SCF complex-mediated degradation of SAS-6 in order to stabilize the cartwheel structure (124, 125). After the cartwheel is well formed, CPAP, CEP135 and γ-tubulin promote the deposition of microtubules around the cartwheel so as to facilitate the elongation of the procentriole (39). However, the extension of the centriole is not infinite. Coiled-coil protein 110 (CP110), most of which is located at the distal end of the centriole, serves to limit further elongation of centrioles (126, 127). Lee et al. found that Plk4 phosphorylated a CP110 on Ser98 and that a fraction of the phosphorylated CP110 was located at the proximal end of two centrioles, suggesting a critical role in assembling centrioles (128). Aberrant activation of Plk4 kinase results in redundant centrosomes (38, 85, 129), which leads to chromosome mismatch, CIN and oncogenesis (113). In addition, it has been shown that cytokinesis defects can also cause chromosomal instability (130, 131). Rosario et al. demonstrated that Plk4 phosphorylated Ect2, a Rho guanine nucleotide exchange factor, which activated Rho GTPase and triggered cytokinesis. In Plk4+/- MEFs, haploid Plk4 levels impair the function of Rho GTPase, resulting in cytokinesis defects, and eventually CIN and tumorigenesis (132). Taken together, normal Plk4 levels ensure the normal replication of the centrioles, thus maintaining cell cycle progression and genomic stability (**Figure 2B**). Plk1, which has been well studied before, has been found to play an important role in cell cycle progression and maintaining genomic stability. However, the mechanism of action is quite different from Plk4, especially in the regulation of centrosomes. Plk1 is a key player in the coordination of the centriole cycle with the cell cycle, by controlling centriole disengagement and maturation but not procentriole assembly (12, 47, 48).

### Plk4 in Tumor Invasion and Metastasis

**Figure 6:**
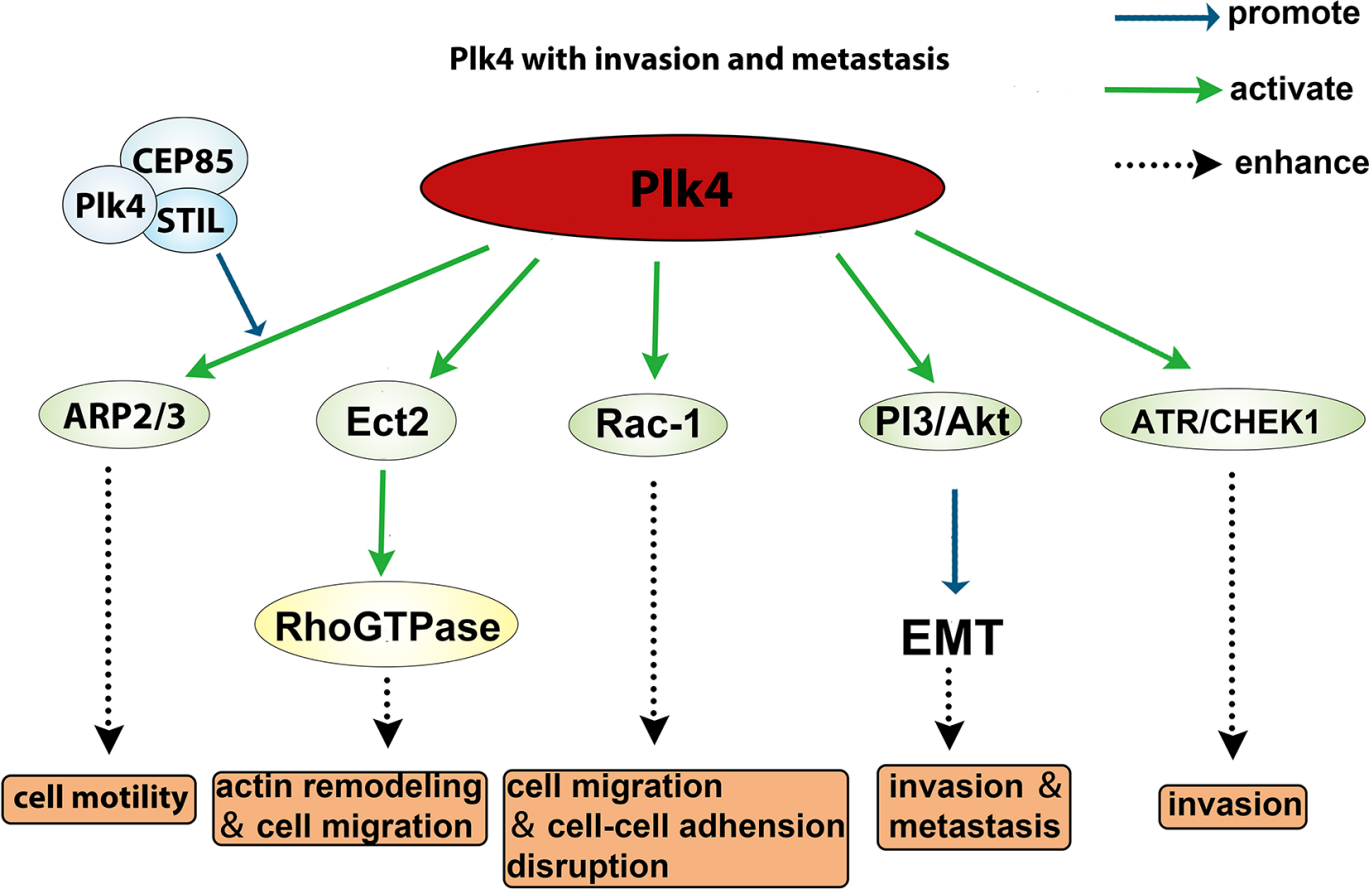
Role of Plk4 in affecting invasion and metastasis.

The role of Plk4 in cancer invasion and metastasis has been well documented. The Arp2/3 complex is essential for generating branched actin networks which are critical for cell motility (133). Kazazian et al. found that Arp2 interacted with the CPB of Plk4 which led to Plk4 phosphorylation of Arp2 on Thr237/238. The phosphorylated Arp2 was activated and promoted cell motility, suggesting that Plk4 facilitates invasiveness and metastasis of cancer cells *via* activating the ARP2/3 complex (134). Liu et al. showed that the cancer cell motility was regulated *via* the interaction between CEP85, STIL, and Plk4, and that downregulation of either CEP85 or STIL led to a reduced level of Arp2 phosphorylation and actin cytoskeleton reorganization (135). In addition, Plk4 was shown to phosphorylate Ect2 (epithelial transforming 2), which in turn activates Rho GTPase to promote actin remodeling and cell migration (43). Moreover, overexpression of Plk4 in MCF10A cells led to activated Rac-1, which disrupted normal cell‒cell adhesion and promoted invasion and metastasis (136). Intriguingly, Tian et al. demonstrated that knockdown of Plk4 reduced epithelial‒mesenchymal transition (EMT) through inhibition of the PI3K/Akt pathway (137, 138). Another group demonstrated that Plk4 mediated activation of ATR/CHEK1 promotes cell proliferation in Hep3B and SMMC7721 cells (73). Collectively, these findings (summarized in **Figure 6**) indicate that Plk4 plays a pivotal role in cancer invasion and metastasis.

### Plk4 in Cell Proliferation

Previous studies showed that Plk4 improve cell proliferation by mediating upstream cell proliferating signals. In U87 cells, ATAD2 was found to upregulate Plk4 leading to promote cell proliferation (63). Knockout of p53 in mice exhibited increased levels of Plk4 which made up for the absence of p53 and induced cell proliferation and squamous cell carcinomas (139). Downregulating both miR-126 (73) and miR-338-3p (74) could increase the Plk4 level, leading to enhanced cell proliferation. Inhibition of Plk4 by small molecules significantly reduced cell proliferation in several cell lines derived from a number of cancers including liver cancer (Huh7 cells) and brain cancer (MON, BT-12, BT-16, G401 cells) (140, 141); YLT-11, a novel Plk4 inhibitor, could suppress breast cancer cell proliferation (MDA-MB-231, MDA-MB-468, BT549, and MCF-7 cell lines) (142); and in human melanoma cell lines (A375 and Hs294T cells), cell proliferation was impaired after centrinone B treatment (11). Taken together, the role of Plk4 in cancer cell proliferation is well established.

### Plk4 in Cell Death

Recent studies showed that Plk4 is an important anti-apoptotic molecule in cancer cells (139, 143, 144). Zhang et al. demonstrated that the Plk4/IKBKE/NF-κB axis was involved in glioblastoma cell survival. In this study, IKBKE was found to interact with and be phosphorylated and activated by Plk4, which subsequently induces transactivation of NF-κB. The activated NF-κB resulted in the transcription of downstream anti-apoptosis genes, thereby resisting cell death (143). In addition, it was shown that overexpression of Plk4 promotes tumorigenesis when p53 is depleted, indicating that Plk4 cooperates with p53 dysfunction in cancer development (139, 144).

### Plk4 in Tumor-Associated Inflammation

The relationship between Plk4 and tumor-associated inflammation has not been widely studied. Yang et al. demonstrated that Plk4 phosphorylated NEK7 at Ser204. The phosphorylated NEK7 impaired its interaction with the NLRP3 inflammasome, preventing NLRP3 from being activated (102). Previous studies showed that inflammasomes are able to activate pro-caspase-1 and mature inflammatory cytokines such as IL-1β and IL-18 (145), which play a crucial role in cancer-related inflammation (146). Further investigations are needed to reveal the role and mechanism of Plk4 in tumor-associated inflammation.

## Plk4 in Human Malignancy

### Plk4 Level in Normal Tissues and Tumor Tissues

**Figure 7:**
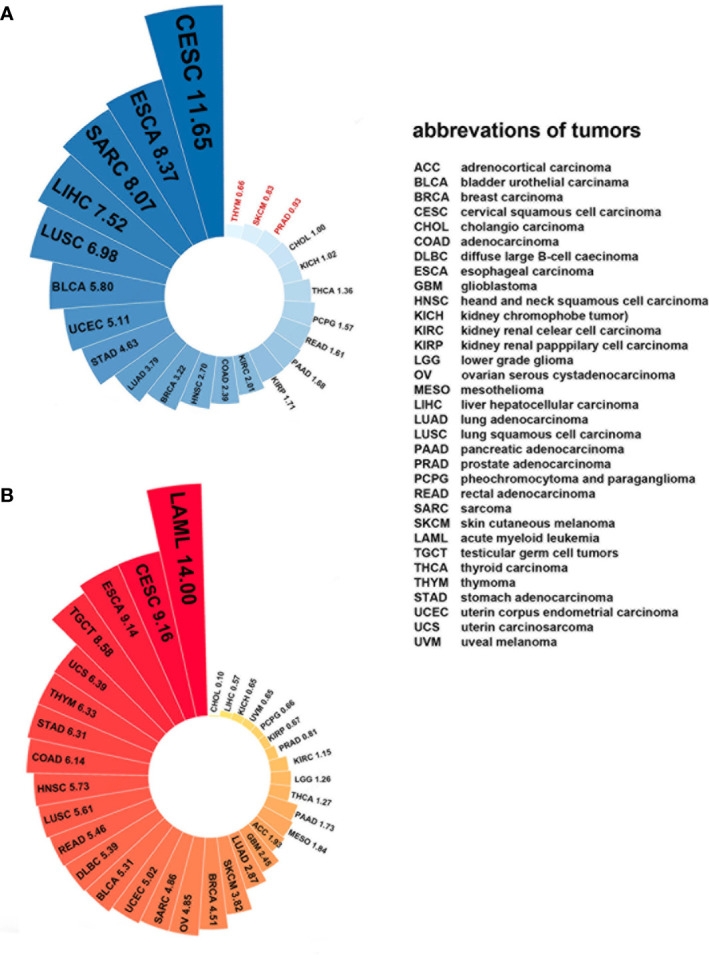
Plk4 mRNA expression in cancer and normal tissues from TCGA database. **(A)** Differences in Plk4 expression between different cancers and surrounding normal tissues, where the values represent the ratio of TPM (transcripts per kilobase per million mapped reads) median between cancer tissues and corresponding normal tissues. It can be seen that in THYM, SKCM, and PRAD, this ratio is less than 1, which means that the median Plk4 expression level in these three cancer tissues is lower than that in corresponding normal tissues. **(B)** Expression level of Plk4 in different cancer tissues, where the value represents the median TPM of Plk4 expression level.

Since Plk4 is a key molecule that regulates mitosis in mammalian somatic cells, its expression levels are closely related to the degree of proliferation of somatic cells. For example, the level of Plk4 is high in the testes because the germ cells are always dividing. Some tissues with less vigorous division, such as epithelial tissues, have lower levels of Plk4. The level of Plk4 was almost undetectable in tissues that were barely renewed, such as cardiac muscle and the lungs (84, 147). Similarly, Plk4 was widely overexpressed in most cancerous tissues than in normal tissues because of the active proliferation and division of cancer cells (**Figure 7A**). Moreover, the expression level of Plk4 varied in different cancers (**Figure 7B**).

### Plk4 and Digestive System Tumors

Hepatocellular carcinoma (HCC) is responsible for 90% of the primary hepatic carcinomas and it is also the fifth most common cancer in men and ninth most common in women worldwide (148, 149). HCC has the second highest mortality rate of any cancer worldwide (150, 151). Both upregulation and downregulation of Plk4 were found in HCC. Liu et al. discovered that lower level Plk4 were associated with advanced stage, high levels of serum alpha fetoprotein (AFP), larger size tumors and shorter overall survival in 246 HCC patients (152). Loss of heterozygosity (LOH) was found in more than half of the HCC samples, in which Plk4 levels were lower than in adjacent normal tissues (132, 153, 154). This was consistent with the previous discovery in which the Plk4 locus on chromosome 4q28 was found to be missing (155, 156). Follow-up studies showed that LOH of Plk4 locus resulted in cell cycle delay (46) and mitotic failure (157), which would promote HCC progression. In addition to gene deletion, Alejandra et al. demonstrated that hypermethylation of the Plk4 promoter in HCC also led to a decrease in Plk4 expression (153). These observations indicated that LOH, as well as epigenetic alternation of the Plk4 gene, reduces expression, which is critical to the development of HCC. However, other studies found that the upregulation of Plk4 was associated with poor prognosis in some HCC samples (73, 140). Meng et al. found that rs3811741, a functional cis-expression quantitative trait loci (eQTL) genetic variant of the Plk4 gene and located in the Plk4 intron, was significantly associated with HCC risk. The high level Plk4 was related to risk allele A of rs3811741 in HCC tissues. The proliferation rate of HCC cells, in which the Plk4 levels were lower than in adjacent normal tissues, was significantly suppressed by a potent selective Plk4 inhibitor CFI-400945 (140, 158). In addition, Bao et al. showed that downregulation of miR-126 in HCC tissues increased the expression level of Plk4 *via* a ceRNA mechanism. The upregulated Plk4 subsequently activated the ATR/CHEK1 pathway which is critical for maintaining genomic stability and promoting tumor progression. Thus, the miR-126/Plk4/ATR/CHEK1 axis is important for the regulation of HCC progression (73). In all, for different reasons, both high and low expression levels of Plk4 have been found in HCC and are associated with clinical parameters, such as prognosis and cancer progression.

Patients with colorectal cancer (CRC) have a poor prognosis; and patients with metastatic colorectal cancer have a low 5-year survival rate is less than 20% (159). Downregulated Plk4 mRNA level was found in CRC and was closely related to GIN (160). On the other hand, the level of Plk4 transcripts in CRC was approximately 1.31 times higher than in adjacent normal tissue, and this number is even much higher in patients over 60 years old because Plk4 expression decreases in normal tissues with age. However, no correlation was shown between Plk4 level and tumor stage (161). Ko et al. found that in colorectal cancer with CIN and microsatellite instability, Plk4 was downregulated (160). Kim et al. reported that CEP131, which is a centriolar satellite protein associated with genomic stability maintenance, is phosphorylated by Plk4 on Ser21 and Thr205, and the phosphorylated CEP131 increases the ability to bind with STIL, causing the stabilization and activation of Plk4. Similarly, an xenograft mouse model generated from HCT116 colon cancer cells revealed that centrosome amplification (CA) together with tumor growth were both dramatically enhanced by CEP131 upregulation (122). This may reveal the important function of Plk4 in regulating GIN. Rosario et al. found that exogenous overexpression of Plk4 in DLD-1 colon cancer cells increased cell mobility and invasion (43); while when p53’s function was impaired, the effect of Plk4 inhibition on tumor growth was significantly reduced (162). This may indicate that Plk4 does not play a significant role in regulating cell cycle progression.

Gastric cancer (GC) is the fifth most common type of cancer in humans and the third leading cause of death in East Asian countries (163). Shinmura et al. showed upregulation of variant 1 of Plk4, a predominant transcript, in various GC cell lines, especially the AGS cell line. Overexpression of Plk4 induces CA and CIN in GC cells (54). Pancreatic cancer (PC) is a fatal malignancy that is predominantly seen in men over 40 years old and has a very aggressive course (164). Plk4-mediated centriole overduplication has been identified as a biomarker of poor prognosis in aggressive PC (160, 165). Lohse et al. generated PC patient-derived xenografts. The size of the tumor and number of tumor-initiating cells could be reduced after CFI-400945 treatment, and thus the overall survival of xenograft models was increased (166). These results suggest that Plk4 inhibitors may be potential drugs for pancreatic cancer.

### Plk4 and Nervous System Tumors

Glioblastoma (GBM) is one of the most serious malignant tumors in adults, with a poor prognosis and a median survival of less than two years (167–169). High expression of Plk4 often occurs in patients with high-grade glioma and is often associated with poor prognosis in TCGA and CGGA datasets (63). GBM standard therapy is often ineffective because of temozolomide (TMZ) and radiotherapy resistance. Enhance of the chemo- and radiotherapy sensitivity might improve the prognosis of GBM patients. Plk4 was shown to phosphorylate IKBKE and thereby induce NF-κB transactivation, which results in enhancing proliferation and chemoresistance. Small molecule Plk4 inhibitor CFI-400945 could largely restore the temozolomide (TMZ) sensitivity. *In vivo* experiments showed that TMZ modestly improved survival and reduced tumor burden in patient-derived xenografts. Combined treatment with TMZ and CFI400945 significantly reduced tumor burden and improved survival. The median survival results showed that the of TMZ and CFI400945 cotreatment group (31.5 days) lived significantly longer than the control (20.3 days), and TMZ (25.7 days) groups (143). These results show that combination of TMZ and Plk4 inhibitor enhances the antitumor effect in GBM. On the other hand, Liu et al. showed that Plk4 induced radio-resistance in GBM, while Plk4 knockdown significantly increased the radiosensitivity of GBM cells. Mechanically, ATAD2-dependent transcriptional regulation of Plk4 enhanced radio-resistance *in vitro* and *in vivo (* 63). Bortezomib was initially approved by US Food and Drug Administration (FDA) for treatment of refractory multiple myeloma (MM). Recent research showed that it is important in the regulation of cell cycle, mitosis, cell viability, proliferation and apoptosis in GBM cell. In addition, the anti-tumor effect of bortezomib was enhanced after Plk4 knockdown in three (LN-18, A172 and LN-229) GBM cell lines and xenograft experiments. Further investigations indicate that this effect may be mediated by the PTEN/PI3K/AKT/mTOR signaling pathway (170). Thus, these findings indicated that Plk4 is a promising therapeutic target for GBM.

Rhabdoid tumors (RT) are highly aggressive and vastly unresponsive types of embryonal tumors (171). Although this tumor can appear anywhere in the body, it occurs most frequently in the central nervous system, where it is known as an atypical teratoid/rhabdoid tumors (AT/RT) (172). Sredni et al. found that Plk4 could drive rhabdoid tumors *via* systematic screening of the kinome. The proliferation, viability and survival of Plk4 CRISPER-mutated rhabdoid cells were significantly reduced *in vitro*. Small molecule Plk4 inhibitor CFI-400945 was also found to significantly abrogated Plk4-induced cell proliferation, and to minimal toxic effects over zebrafish larvae (141). The efficacy of CFI-400945 in orthotropic RT xenografts were examined by Tomita et al. and they found that CFI-400945 could mediate the generation of polyploidy makes RT cells more sensitive to DNA-damaging drugs which has the therapeutic effect of cytotoxicity (173). Medulloblastoma (MB), arising in the cerebellum, is the most common pediatric high-grade brain tumors and the major cause of morbidity and mortality in pediatric oncology (174, 175). A number of studies have shown that the relative expression of Plk4 in MB was 39.66 times that in normal cerebellum (176, 177). The above results showed that the proliferation, survival, migration, and invasion of RT cells were significantly reduced with Plk4 inhibitor CFI-400945. Sredni et al. also demonstrated that after treated with CFI-400945, MB cells exhibited induced apoptosis, senescence and polyploidy, thereby become more susceptible to DNA-damaging agents in orthotropic xenograft model. Based on these findings, Plk4 inhibitors may be ideal candidate drugs for RT and MB therapeutics solo or in combination with cytotoxic agents (173).

Neuroblastoma (NB) accounts for disproportionate morbidity and mortality among the cancers of childhood, with primary and metastatic tumors in the central nervous system (178, 179). A higher level of Plk4 has been found in NB both primary and metastatic and is associated with a poor prognosis, which suggested that Plk4 could be a potential tumor-promoting factor of NB (177, 180). Zhang et al. found that there was a negative correlation of expression between Plk4 and miR-338-3p in NB tissues. They further verified Plk4 as a target gene of miR-338-3p in NB cells. Functional analyses showed that miR338-3p upregulation could inhibit the expression of Plk4 and phosphorylation of Akt (74). Additionally, in SK-N-BE (2) NB cells, downregulation of Plk4 *via* shRNA suppressed EMT and promoted apoptosis through the PI3K/Akt signaling pathway (138, 181). These results revealed that targeting Plk4 as a promising therapeutic regimen for pediatric embryonal tumors is suitable for further investigation.

### Plk4 and Reproductive System Tumors

Breast cancer (BC) accounts for one of the largest percentages of cancer-related deaths among women in the world (182–184). Although great progress has been made in the diagnosis and treatment of this disease, the overall prognosis of BC, especially metastatic BC, remains poor (185). Plk4 was found to be overexpressed in BC tissues (186, 187), and high expression levels of Plk4 in BC were found to be associated with poor prognosis and disease aggressiveness (188, 189). Mi et al. found that overexpression of Plk4 and transcription factor E2F were strongly correlated in breast cancer. Excessive E2F upregulated Plk4 expression at a transcriptional level, leading to centrosome amplification and CIN in MCF10A mammary epithelial cells (59, 182). In addition, Plk4 cooperates with NEK2, a regulatory kinase in the centrosome, to promote BC progression (190). Overexpression of one or both these kinases (Plk4 and NEK2) led to poor prognosis in patients who were treated with trastuzumab and tamoxifen (191). These results showed that NEK2, in synergism with Plk4, may stimulate breast tumorigenesis. Godinho et al. showed that centrosome amplification triggered by Plk4 enhanced the invasiveness of BC cells, which is similar to that induced by overexpression of ERBB2 (136). Consistently, Swallow et al. found that Plk4 depletion impairs invasion of murine embryonic fibroblasts and suppresses invasion *via* cytoskeletal reorganization and development of polarity in MDA-MB231 BC cells (43). A recent study demonstrated that YLT-11 (a novel Plk4 inhibitor) treatment caused abnormal centriole numbers and defective mitosis in BC cells, especially for triple-negative breast cancer (TNBC) cells. Moreover, YLT-11 dramatically delayed tumor proliferation in orthotropic BC mouse models generated with MCF-7, MDA-MB-468, and MDA-MB-231 cells (142). Another Plk4 inhibitor CFI-400945 also triggered mitotic defects in MDA-MB-468 and MDA-MB-231 cells by the dysregulation of centriole number and the induction of polyploidy (158) and inhibited tumor growth in xenograft mouse model established with MDA-MB-468 cells (192). These data corroborate that Plk4 may be a promising target for the treatment of BC and is of value for further study.

Cervical cancer (CC) is the fourth most common cancer among women globally (193). Plk4 was demonstrated to promote cervical tumorigenesis. Previous studies have established a close relationship between human papillomavirus (HPV, oncogenic virus) and cervical cancer (194–196). It has been shown that the oncoproteins such as E6 and E7 encoded by HPV disrupted mitosis and induced GIN and CC tumorigenesis (197, 198). Further investigations demonstrated that the HPV-16 E7 increased the expression of Plk4 by impairing the repression effect of DREAM on the Plk4 promoter in human cervical cancer cell lines. A higher level of Plk4 triggered centrosome amplification and tumorigenesis (65, 199). These results emphasize the role of Plk4 in HPV-associated cervical carcinogenesis. In addition to HPV, Chlamydia trachomatis infection is also important to the development of CC (200, 201). Chlamydia trachomatis infection leads to an abnormal number of immature centrioles and mitotic failure in HeLa cells. This process relies on the kinase activity of Plk4 and CDK2, which are required for formation of a daughter centriole from a template centriole during S-phase (202). Taken together, these findings indicate that HPV- and Chlamydia trachomatis-caused dysregulation of Plk4 plays a pivotal role in the development of CC.

### Plk4 and Lung Cancer

Lung cancer is one of the most common causes of cancer death in humans and is also the most common cancer (203). About 85% of lung cancer is non-small-cell lung cancer (NSCLC). Lung adenocarcinoma (LAC) and lung squamous cell carcinoma (LSCC) make up 38.5% and 20% of NSCLC, respectively (204, 205). The retrospectively analyzed data from 560 surgical NSCLC patients showed that the high level of Plk4 was found to be correlated with larger tumor size, wider lymphatic metastasis and higher TNM stage. Patients with the high level of Plk4 exhibited the poor prognosis (206). The above data revealed the correlation of Plk4 with clinicopathological features and prognosis in NSCLC patients. Kawakami et al. showed that levels of Plk4 were higher in LAC and LSCC than in adjacent normal lung tissues, and that CFI-400945 treatment led to polyploidy and apoptosis in murine and human lung cancer cells by triggering multipolar mitotic defects (207). Plk4 inhibition holds promise in lung cancer therapy either as a single agent or when combined with an agent that deregulates mitosis. DNA Polymerase Theta (POLQ) is a DNA polymerase involved in translation DNA synthesis (TLS) and DNA double-strand break (DSB) repair. Moreover, elevated expression levels of Plk4 were largely correlated with overexpression of DNA polymerase PQLQ at both mRNA and protein in LAC and co-expression of Plk4 and POLQ induced more significant centriole overduplication than the expression of either one alone, suggesting the potential cooperation of Plk4 and PQLQ in LAC oncogenesis (208).

### Plk4 in Other Carcinomas

Osteosarcoma is the most common primary bone tumor with a high mortality rate in adolescents (209, 210). Li et al. demonstrated that p53 could recruit HDAC transcriptional repressors to Plk4 promoters so as to play the role of transcriptional repressor, and Plk4 inhibition would also lead to p53-mediated apoptosis. These results indicated that Plk4 appears to be a promising cancer target for p53-dependent induction of apoptosis in osteosarcoma (68). In addition, abnormal expression of the Plk4 in cancer is often correlated with aberrant promoter methylation. The promoter region of Plk4 was found to be hypermethylated in Saos-2 cells when the cells were treated with oxygen deprivation, resulting in a decreased expression level of Plk4. External environmental stimuli, oxidative stress, would induce changes to the promoter methylation of the Plk4 resulting in changes in expression. Moreover, a higher concentration (≥200 nM) of CFI-400945 resulted in a decrease in the average centriole number of asynchronous U2OS cells, while a lower concentration (10–100 nM) of CFI-400945 resulted in an increase in the average centriole number relative to control cells (158). These results suggest that an aberrant level of Plk4 plays a role in the genesis of osteosarcoma.

Melanoma is the most aggressive form of skin cancer, and excessive sun exposure is still considered the major environmental risk factor. Cutaneous melanoma is one of the most invasive human cancers and causes most of the skin cancer-related deaths (211). Compared with normal melanocytes, Plk4 was found to be dramatically upregulated in melanoma. The higher the Plk4 expression level was, the worse the survival rate was, but the difference was not significant in the TCGA melanoma dataset. Additionally, Plk4 overexpression was associated with centriole overduplication in tissue microarray of human melanomas, which means that the expression of Plk4 is the main driver of melanoma centriole duplication. Further investigation demonstrated that centrinone B, a selective Plk4 inhibitor, depleted centrioles and induced apoptosis in A375 and Hs294T melanoma cells, suggesting Plk4 is a potential biomarker and drug target in melanoma (11).

## Inhibitors of Plk4 and Their Effects in Cancer Treatment

Plk4 has been reported to be widely overexpressed in tumor samples from cancer patients, and its overexpression has been shown to be a biomarker for the poor prognosis of many human cancers. Therefore, Plk4 has been repeatedly proposed as a particularly attractive target for the discovery of anticancer drugs. Several small molecule Plk4 inhibitors have been identified and are summarized in **Table 1**.

**Table 1:** Properties of Plk4 inhibitors.

Inhibitors	CFI-400945	CFI-400437	Centrinone	Centrinone B	YLT-11
**Important site involved in binding to Plk4**	Leu-17/ Val-25/ Ala-38/Cys-91/Arg-98/ Leu-142	Leu-17/ Gly-18/ Val-25/ Ala-38/ Cys-91/ Arg-98/ Leu-142	Val-25/ Lys-40/ Glu-89/ Cys-91/ Leu-142	Val-25/ Lys-40/ Glu-89/ Cys-91/ Leu-142	Phe-23/ Glu-90/ Met-91/ Cys-92/ His-93/ Leu-143/ Gln-160
**IC50 (nM)**	2.8	1.55	2.71	8.69	22
**Structure**	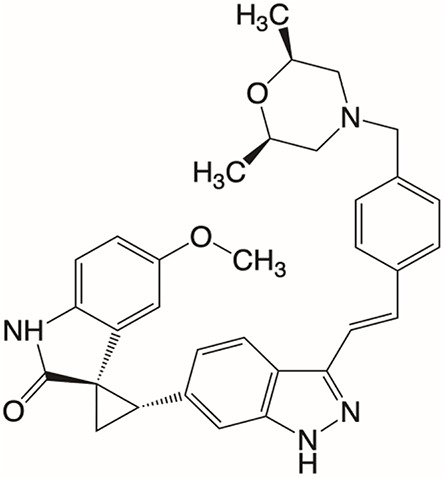	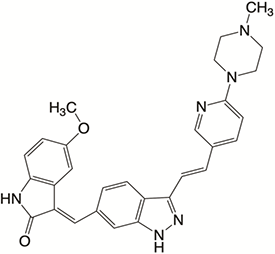	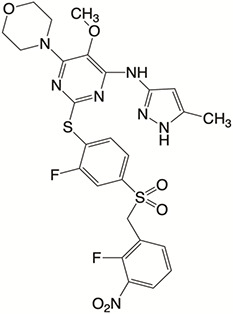	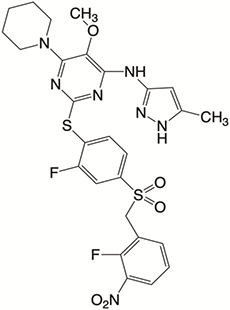	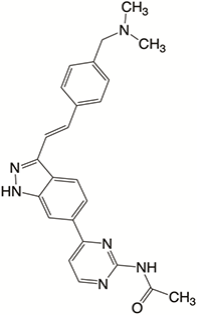

There is only one druggable domain of Plk4 that have been researched extensively: the catalytic domain, namely kinase domain (KD). CFI-400945 was the first orally available potent Plk4 inhibitor and it binds to the ATP-binding pocket of Plk4 KD and the IC50 of CFI-400945 against Plk4 is 0.6 nM, which is lower than those against other Plk family members (Plk1>50,000 nM; Plk2>50,000 nM; Plk3>50,000 nM) (158, 212). To be more specifically, CFI-400945 inhibits autophosphorylation of Plk4 at Ser-305 which is a critical autophosphorylation site for Plk4 activation. Manson et al. showed the selective antitumor activity of CFI-400945 in breast cancer cells in which Plk4 was overexpressed (158). Further investigations proved the remarkable antitumor effects of CFI-400945 in some kinds of cancers, including pancreatic cancer (166), lung cancer (207), liver cancer (140) and breast cancer (192). Interestingly, due to the self-regulating function of Plk4, CFI-400945 has a double effect on the number of centrioles by inhibiting Plk4: high concentration CFI-400945 inhibits the generation of centrioles, while low concentration CFI-400945 increases the number of centrioles possibly because partial Plk4 inhibition still phosphorylate downstream substrates (158). Moreover, CFI-400945 was shown to have synergistic effects with some chemotherapeutic drugs. For example, previous studies by our group have confirmed that after CFI-400945 treatment, GBM cells became more sensitive to TMZ (143). Furthermore, CFI-400945 sensitized RT and MB tumors to DNA damage drugs-induced cell death, and/or cell cycle arrest (141). Currently, there are five CFI-400945 clinical trials in various human malignancies (**Table 2**). We are anxiously awaiting the results of the clinical trial in the hope of having an advisory opinion on our research, i.e., whether controlling the number of centrioles can significantly inhibit the development of cancer.

**Table 2:** Clinical trials of CFI-400945.

Trial ID	Phase	Disease	Treatment regimes	State	Period	Gender，age and numbers of participants			Purpose	Outcome Measures
NCT04176848	2	Advanced Triple Negative Breast Cancer	cycle1: orally on Days 1-7 (then 7 days off) and on Days 15-21 (then 7 days off) cycle2: orally once daily (28 day cycles)	Not yet recruiting	November 2019 —December 2020	Female	»18	28	Treatment	**Primary Outcome Measures (time frame：24 months)** Objective response rate of CFI-400945 given with durvalumab using RECIST 1.1
NCT03187288	1	Acute Myeloid Leukemia; Myelodysplastic Syndromes; Relapsed Cancer; Refractory Cancer	Orally, 64, 72, 96, 128, 176, or 224 mg/ day, every day until intolerable side effects or disease progression	Recruiting	May, 2018 —April, 2022	ALL	»18	48	Treatment	**Primary Outcome Measures (time frame：5 years)** 1.To assess the safety of CFI-400945 Fumarate 2.Highest tolerated dose of CFI-400945 fumarate 3.Recommended phase 2 dose of CFI-400945 fumarate
NCT01954316	1	Advanced Cancer	Orally, 3, 6, 11, 16, 24, and 32 mg/day	Recruiting	March 2014 —October 2021	ALL	»18	48	Treatment	**Primary Outcome Measures (time frame：up to 2 years)** Highest dose level that does not lead to unacceptable toxicity in two or more patients in a dosing group over a range of doses and schedules
NCT03624543	2	Advanced/Metastatic Breast Cancer	Cycle1 64 mg orally day 1–7 every 14 days Cycle2 64 mg orally daily for 28 days	Recruting	December 12, 2017 —December 31, 2021	Female	»18	72	Treatment	**Primary Outcome Measures (time frame： 2 years)** Objective response defined by RECIST 1.1
NCT03385655	2	Prostate Cancer	64 mg orally day 1–7 every 14 days	Recruting	December 12, 2017 —December 31, 2021	Male	»18	500	Treatment	**Primary Outcome Measures (time frame： 2 years)** Clinical benefit rate defined as proportion of patients who had PSA decline ≥ 50%, complete or partial objective response, or Stable disease for ≥ 12 weeks.

Similar to CFI-400945, CFI-400437, an indolinone-derived ATP-competitive kinase inhibitor, has an IC50 value of 1.55 nM for Plk4 and exhibits low inhibitory activity against other members of the Plk family (213). It has been shown that CFI-400437 significantly inhibits Plk4 activity and tumor growth in breast cancer, both in a cell culture and in a xenograft mouse tumor model (213, 214). Poor brain exposure and selectivity are also drawbacks of this inhibitor.

In terms of selectivity, CFI-400945/CFI-400437 was not only effective in inhibiting Plk4, but also active against AURKB, TRKA, TRKB, and Tie2/TEK (215). Thus, centrinone/centrinone B, two more specific compounds which also bind to the ATP-binding pocket of Plk4 KD were developed. Centrinone (IC50 2.71nM) and centrinone B (IC50 8.69nM) are two reversible and highly selective Plk4 inhibitors which lead to total but reversible centrosome loss (214, 216). They can suppress centriole duplication and centriole assembly so as to reduce centrosomes. HeLa cells treated with centrinone led to p53-mediated G1 cell cycle arrest. Human melanoma cell lines (A375, Hs294T, G361, and SK-MEL-28) treated with centrinone B treatment exhibited a decrease in cell viability and an increase in apoptosis (11). However, in some cancer cell lines (HeLa and NIH/3T3 cells), centrinone therapy inhibited cell proliferation unrelated to centrosomal loss, suggesting an inherent “set point” for centrosomal numbers. Thus, centrosome depletion was insufficient for cancer therapy and should be combined with other targeted therapy drugs (216). Another drawback of Centrinone/Centrinone B is that it is very difficult for this compound to penetrate the blood-brain barrier, so it may not be able to treat tumors in the brain (214).

YLT-11 is a newly designed selective Plk4 inhibitor (IC50 22nM). Lei et al. showed that YLT-11 considerably inhibited breast cancer cell proliferation and led to maladjustment of centriole replication and mitotic defects, which increase the sensitivity of tumor cells to chemotherapy. In human breast cancer xenograft models (MCF-7, MDA-MB-468, and MDA-MB-231), mice treated with YLT-11 exhibited dramatically decreased tumor growth. These results suggested that YLT-11 is a promising drug for breast cancer patients (142). And we look forward to a clinical trial about YLT-11 soon.

## Challenges and Future Prospects

Plk4 is a structure-specific member of the Plk family and has been identified as a key regulator of centriole replication in previous studies. Abnormal expression levels are also widely found in cancer and are closely associated with poor prognostic clinical indicators. Therefore, Plk4 has received extensive attention as a biomarker and a target for cancer treatment. However, there still are challenges for the strategy for targeting Plk4, because currently the etiology of Plk4 in tumorigenesis and cancer development has not been fully understood. Although the increase and decrease of Plk4 expression have been reported in many tumor types, no Plk4 recurrence/driver mutation was found in the genome sequencing of cancer cells. It is not clear whether changes in Plk4 levels are the cause of tumor development or progression. Inhibition of Plk4 levels may lead to the failure of centrosomes replication, while overexpression of Plk4 will lead to the formation of redundant centrosomes which would drive centrosomal amplification and subsequent GIN. For example, Plk4 heterozygous mice are predisposed to tumorigenesis (46) and overexpression of Plk4 in Drosophila neuroblasts promotes transformation (217). Furthermore, Plk4 is a low abundance enzyme that phosphorylates itself *via* the formation of Plk4 homodimers to promote its own destruction. It has been reported that complete inhibition of Plk4 by CFI-400945 treatment led to an increase in Plk4 level and a failure of centriole duplication, but lower doses of CFI-400945 will promote an increase in centriole number (158). The bimodal effect of CFI-400945 concentration on centriole number is very surprising, which may be explained by formation of heterodimers between kinase inactive and catalytically-active Plk4, under that condition, kinase inactive Plk4 is unable to trans-autophosphorylate and destabilize wild type Plk4, leading to an increase in the abundance of the wild type kinase that results in centriole overduplication. Nevertheless, Plk4 is still a potent therapeutic target, because centrosome amplification has a close correlation with tumorigenesis. A recent report has shown that a single nucleotide polymorphism (SNP) rs2305957 (4q28.1), located in the region of chromosome 4 and encompass the Plk4 gene is associated with aneuploidy of mitotic origin in early human embryonic development, and that this mutation can lead to embryonic death (218). The haplotype associated with SNP rs2305957 lies in a region of low recombination spanning more than 600 Kbp of chromosome 4. This region contains genes INTU, SLC25A31, HSPA4L, Plk4, MFSD8, LARP1B, and PGRMC2. Plk4 is the most attractive aneuploidy related candidate, because it is a master regulator of centriole replication, a key component of the centrosomal cycle, is essential in mediating the formation of bipolar spindles during the first cell division in mouse embryos. On the other hand, whether the location of Plk4 in the chromosome make its function specially is not clear, (see chromosome locations of Plks: Plk1: 16p12.2, Plk2: 5q11.2, Plk3: 1q34.1, Plk4: 4q28.1, and Plk5: 19p13.3). Since both up- and down-regulation of Plk4 have the potential to induce CIN. Abnormal expression of Plk4 has been observed in several cancers, it would be an interesting topic whether SNP or mutation of Plk4 gene in cancer will cause more frequency in aneuploidy. Additionally, Zhang et al. found that SNP rs2305957 is associated with early recurrent abortion (219). Due to the close association between embryonic development and tumorigenesis in terms of genes, proteins, metabolic levels and important biological behaviors (108, 220), we have a hypothesis that SNP rs2305957 might be used as a biological biomarker for activation of Plk4 gene in cancer development. This could be a very interesting research project, because uncovering the association of SNP rs2305957 and dysregulated Plk4 in cancer might lead to development of a novel cancer biomarker or therapeutic target.

From a therapeutic point of view, aneuploidy and GIN are characteristics that distinguish cancer cells from normal cells and represent tumor-specific weaknesses that can be exploited. Plk4 is a regulator of centrosome duplication. Plk4 overexpression induces CA and may promote carcinogenesis, suggesting that Plk4 is a therapeutic target for cancer. In support of the therapeutic potential of such strategies, some inhibitors against Plk4 are currently undergoing basic research and clinical trials. The main problem currently associated with Plk4 inhibitors is the lack of specificity. Further studies are needed to identify new compounds with higher potency and specificity as well as better pharmacokinetic properties. Broadly, two major sites of Plk4 can be potential targets: one is the adenosine-5’-triphosphate (ATP)-binding site in the KD and the other is PBD domain. CFI-400945 fumarate is a first, oral selective ATP competitive inhibitor of Plk4. Although the only inhibitor in clinical trials, CFI-400945 exhibits considerable antiproliferative actions in cancer, it also has activity against AURKB, TRKA, TRKB, and Tie2/TEK (213). Therefore, we suspect that the phenotypic anticancer effect of CFI-400945 may also be the result of inhibiting other kinases. It is urgent to design a more selective Plk4 inhibitor and here are a few prospections. When Plk4 was first synthesized, it was in a monomer state, and it needed to form a homodimer of PB1-PB1&PB2-PB2 with another Plk4 monomer to be activated. This phenomenon was not found in other members of Plk families, Aurora kinase families and TRK family. So, we think it’s a very specific phenomenon for Plk4 (221). This gives us a direction for the design of a specific inhibitor which blocks the formation of Plk4 homodimer. Moreover, the PB1-PB1&PB2-PB2 homodimer formed by Plk4 also provides a platform for the interaction between Plk4 and other molecules. If this homodimer can be blocked, the bioactivity of Plk4 can be greatly reduced. Based on this hypothesis, we hypothesized that Plk4-specific PB1-PB1&PB2-PB2 homodimer might serve as potential inhibitor design targets. According to this, another Plk4 specific fragment PB3 may also serve as a potential inhibitor design target. Compared to other Plks, it is still not fully understood that (1) What is the difference of PB3 in Plk4, compared to the other Polo-box domain such as PB1 and PB2? (2) Does PB3 in Plk4 makes some special biological functions which are not so apparent in other Plks? (3) Is there a possible to develop a high selective Plk4 inhibitor *via* targeting PB3 in Plk4? To this end, to design a highly selective Plk4 inhibitor vis targeting its PB3 domain is required.

In the precision medicine, protein targeted therapy is a significant therapeutic tool. Currently, there are two major cancer treatment methods for targeting proteins: small molecule inhibitors (222) and monoclonal antibodies (223). Although encouraging progress has been made in clinical treatment, these treatment schemes will eventually produce drug resistance (224). The mechanism of drug resistance is very complex, and it is also the focus of current research. Epidermal growth factor receptor (EGFR), a membrane protein receptor, its mutations or amplification are the major driving factors of cancer, especially in NSCLC. And the EGFR tyrosine kinase inhibitors (EGFR-TKIs, e.g., gefitinib and elrotinib) have been widely used for clinical treatment (225). However, patients eventually develop resistance. The mechanisms of resistance include secondary mutation of targeted protein (226), activation of alternative pathways (227), aberrance of the downstream pathways (228), impairment of the EGFR-TKIs-mediated apoptosis pathway (229), ATP binding cassette (ABC) transporter effusion (230), etc. At present, immunotherapy is a very promising approach for cancer treatment. In cancer-immunity cycle, immune checkpoint PD1 and its ligand PDL1 play an important role in tumor resistance to immune-induced apoptosis and promotion of tumor progression. Targeted therapy using monoclonal antibodies against PD1/PDL1 axis can effectively block its tumor-promoting activity, and has achieved satisfactory clinical results. However, resistance can also develop in patients treated with PD1/PDL1 antibody (231, 232). And the mechanisms of resistance mainly include insufficient tumor immunogenicity (233), disfunction of major histocompatibility complexes (MHCs) (234), irreversible T cell exhaustion (235), primary resistance to IFN-γ signaling (236), and immunosuppressive microenvironment (237). Thus, treatments targeting protein alone may also eventually develop resistance.

Therefore, the development of a therapeutic regimen that can be combined with Plk4 protein targeted therapy is critical. Plk4 inhibitors (CFI-400945) has been shown in several studies to be synergistic with the chemotherapy drugs, antagonism, and irradiation in cancers. Thus, the role of Plk4 inhibition should be further explored in combination with other anticancer agents. Morris et al. found that the breast cancer cell caused by Plk4 overexpression was more sensitive to the treatment of Stattic and BBI-608, both of which were inhibitors of centrosome clustering regulator STAT3 (238). Raab et al. also discovered a centrosome clustering inhibitor, GF-15 could significantly suppresses Plk4 overexpressed multiple myeloma cells (239). These two findings may provide the possibility of a combination of Plk4 inhibitors and centrosome clustering inhibitors. Clinical testing by adding the Plk4 inhibitor to immune checkpoint inhibitors therefore represents an attractive strategy in future.

Additionally, it is widely known that Plk4 is a key regulator of centriole replication. In the previous report, we know that Plk4 phosphorylates STIL on its STAN domain, thus promoting SAS-6 recruitment. The interaction between STIL and SAS-6 promoted the formation of nine-fold cartwheel of centrioles. So instead of designing inhibitors against Plk4 itself, targeting the interaction of Plk4 with the key binding partners can also be approached. We inhibit Plk4 mainly to inhibit overduplication of centrosomes and prevent normal cells from becoming cancerous. Therefore, we can look for a factor that interacts with Plk4 and is a key factor for centriole replication, and then block this interaction specifically, thus inhibiting the centrosomal amplification effect. Certainly, we should also be concerned about combination therapy with Plk4 inhibitors and other drugs.

## Conclusion

Aberrant expression of Plk4 has frequently been detected in various human malignancies and was identified as a key driver of oncogenesis. In this review, we summarized the regulation of Plk4 at the DNA, RNA, and protein levels, as well as the roles of Plk4 in cellular processes that are involved in human cancer. Abnormal expression of Plk4 in various tumors and the underlying potential mechanisms were also summarized. Several small-molecule inhibitors of Plk4 have been identified, and some of them are currently in clinical trials. Preclinical data showed that targeting Plk4 is a promising therapeutic intervention in a subset of human cancers that express a high level of Plk4. Plk4 inhibitors also exhibited synergy with some chemotherapy drugs. In this review, we reasonably propose several possible Plk4 inhibitor designs, including targeting specific biological behaviors and domains of Plk4, as well as downstream targets that interact with Plk4. We hope these conjectures will help in the future design of more potent and selective Plk4 inhibitors. Future investigations are needed to better our understanding of the mechanisms of Plk4 in cancer development and the efficacy and potential resistance of Plk4 inhibitors.

## Author Contributions

LH conceived the review. XZ and CW drafted the manuscript and revised it before submission. CW and HL collected the references. All authors contributed to the article and approved the submitted version.

## Funding

This work was supported by the grants (Nos. 81773187 and 81572496) from the National Nature Science Foundation of China. Support was also received from the Tianjin High School Program for Young and Middle-aged Talents Backbone and the Tianjin Young Medical Talents Program.

## Conflict of Interest

The authors declare that the research was conducted in the absence of any commercial or financial relationships that could be construed as a potential conflict of interest.
